# Potential of the Novel PTA Score to Identify Patients with Peritonsillar Inflammation Profiting from Medical Treatment

**DOI:** 10.1155/2018/2040746

**Published:** 2018-05-28

**Authors:** Christoph Spiekermann, Johannes Roth, Thomas Vogl, Markus Stenner, Claudia Rudack

**Affiliations:** ^1^Department of Otorhinolaryngology, Head and Neck Surgery, University Hospital Münster, Münster, Germany; ^2^Institute of Immunology, University Hospital Münster, Münster, Germany

## Abstract

Peritonsillar inflammation is a common characteristic of both peritonsillar abscess (PTA) and peritonsillitis (PC). The aim of the present study was to apply the PTA score as an objective criterion to identify patients with peritonsillar inflammation (PI) who might profit from medical treatment. Hence, the recently developed PTA score was applied retrospectively on patients suffering from acute tonsillitis, peritonsillitis, and peritonsillar abscess. Analysis of the clinical data, the follow-up, and the initial PTA score was performed. Patients with peritonsillar inflammation show significant higher PTA score values compared to patients with acute tonsillitis without peritonsillar inflammation and healthy controls. Patients with a PTA score ≤ 2 profited from medical treatment consisting of antibiotics in 92.3% of the cases. In 89.2% of the patients with a PTA score > 2, pus was detected during abscess relief. Patients with peritonsillar inflammation who profited from medical treatment had significantly reduced PTA score values and a reduced duration of hospitalization compared to the patients with abscess relief. Thus, the PTA score has the potential as an objective criterion to identify patients with peritonsillar inflammation profiting from medical treatment. Hence, application of the PTA score helps to determine an optimal, individualized treatment approach and might reduce utilization of medical resources.

## 1. Introduction

As a part of Waldeyer's ring, the palatine tonsils are related to the mucosa-associated lymphatic tissue (MALT) and play an important role in nasopharyngeal immunity particularly in children. Although the function of the tonsils in adults remains controversial, a pivotal role as first-line host defense against pathogens seems plausible because of a high density of lymphocytes in tonsil parenchyma [1]. Acute infection of the tonsils is a very common reason for otorhinolaryngological consultation predominantly of young children and young adults. Furthermore, the tonsils can be a trigger for severe head and neck infection with life-threatening complications such as sepsis or airway obstruction [2–4]. The peritonsillar abscess (PTA) is the most common deep head and neck infection that affects patients of all ages and results in numerous outpatient visits and hospitalizations [5]. The favored treatment is antibiotic therapy combined with abscess drainage. Abscess drainage takes the form of needle aspiration, intraoral incision and drainage, or quincy tonsillectomy. Nevertheless, there is no high-quality evidence concerning the effectiveness of these procedures, and correctly identifying which patients would benefit from surgical therapy remains challenging [6]. Furthermore, indications of abscess tonsillectomy became controversial over the past years because this procedure is associated with an increased risk of spread of infection and postoperative tonsillar bed bleeding [3, 7]. Medical therapy has been described to be as successful as initial surgical treatment in patients with less severe symptoms of peritonsillar abscess [5]. Especially, a small group of patients with symptoms suspicious of PTA but no obvious accumulation of pus profits from medical treatment and this entity is called peritonsillar cellulitis (PC) or peritonsillitis [8]. Similarity of the PTA and the PC group is the existence of a peritonsillar inflammation (PI). However, an objective criterion to identify patients with PI who might profit from medical treatment still remains desirable [5].

Recently, we could identify the complex S100A8/A9 to be a helpful objective marker to identify patients with PTA and to differentiate between PTA and PC [9]. S100A8 and S100A9, which are also known as myeloid related proteins (MRP) 8 and MRP14 or calprotectin, are related to the danger-associated molecular patterns (DAMP) and show typically proinflammatory activities in many mouse models of inflammation [10–13]. Predominantly expressed by neutrophil granulocytes and monocytes, S100A8/A9 activates, for example, leukocytes via Toll-like receptor 4 (TLR-4) pathway and increases transmigration rates, phagocytosis, and inflammatory processes [14].

By addition of one point for each symptom like trismus, halitosis, uvula edema, unilateral swelling of the arched palate, and one point for S100A8/A9 levels in the sera or in saliva over the cut-off values (2550 ng/ml, 8180 ng/ml, resp.), a PTA score with a range from a lowest value of 0 to a highest of 6 was developed to identify patients with PTA [9]. Hence, it was the aim of this retrospective study to analyze the new developed PTA score and its value to identify patients with peritonsillar inflammation profiting from medical treatment.

## 2. Material and Methods

### 2.1. Patients and Healthy Controls

This retrospective study included 75 patients who were treated in the department of otorhinolaryngology, University Hospital Münster between February 2015 and February 2017: 24 patients with acute tonsillitis without peritonsillar inflammation (AT) (11 males, 13 females, median age 25 years, (range from 13–65 years)) and 51 patients suffering from peritonsillar inflammation (PI) (26 males, 25 females, median age 32 years (range: 7–83 years)) (Table 1). Peritonsillar abscess was diagnosed by detection of pus during needle aspiration, abscess drainage in local, or tonsillectomy in common anesthesia. If no pus was detectable and the patient profited from medical treatment, the diagnosis peritonsillitis was considered. All patients received medical treatment consisting of antibiotics independent of further procedures. Common antibiotics for intravenous application were penicillin, amoxicillin, amoxicillin/clavulanic acid, ampicillin/sulbactam, or clindamycin in cases of penicillin allergy. Documentation of symptoms and acquisition of saliva and serum samples were performed during initial outpatient consultation. Healthy volunteers (*n* = 15, median age 30.0 years (range: 21–59)) without any history of acute or recurrent tonsillitis served as controls. The study was approved by the institutional ethics committee, and written informed consent was obtained from all subjects.

### 2.2. S100A8/A9 Sandwich ELISA of Sera and Saliva Samples

Serum samples were centrifuged at 3000 rpm for 10 minutes within 2 hours after acquisition, and supernatant was stored at −20°C until analysis. Saliva acquisition was performed with untreated Salivette® (Sarstedt, 51.1534) as described in the manufacturer's datasheet or with collecting saliva in a 50 ml Falcon tube and centrifugation at 1000*g* for 15 minutes. Supernatant was stored at −20°C. S100A8/A9 concentrations were measured with a sandwich enzyme-linked immunosorbent assay using our ELISA protocol for human S100A8/A9 as described earlier [15].

### 2.3. PTA Score

By addition of one point for each symptom like trismus, halitosis, uvula edema, unilateral swelling of the arched palate, and one point for S100A8/A9 levels over the cut-off value of 8180 ng/ml in saliva and over the cut-off value of 2550 ng/ml in serum, a PTA score value for the patients was calculated as described earlier [9]. The PTA score value (*S*_PTA_) ranges from a minimum of 0 to a maximum of 6. A cut-off value of *S*_PTA_ = 2.5 for the existence of PTA was determined.

### 2.4. Statistical Analysis

Results are mean values ± standard error of the mean (mean ± SEM). Student *t*-test was used to detect significant differences in parametric results, Mann–Whitney *U* test was performed to analyze differences between nonparametric groups, and Kruskal-Wallis test was conducted to analyze difference between multiple independent groups. *P* values < 0.05 were considered to be significant. Significant results are marked with asterisks (^∗^*P* < 0.05, ^∗∗^*P* < 0.01, and ^∗∗∗^*P* < 0.001). Statistical analyses and creation of figures were performed with IBM® SPSS® Statistics 24 and SigmaPlot® 12.

## 3. Results

### 3.1. Classification of Patients Based on PTA Score Values

**Figure 1:**
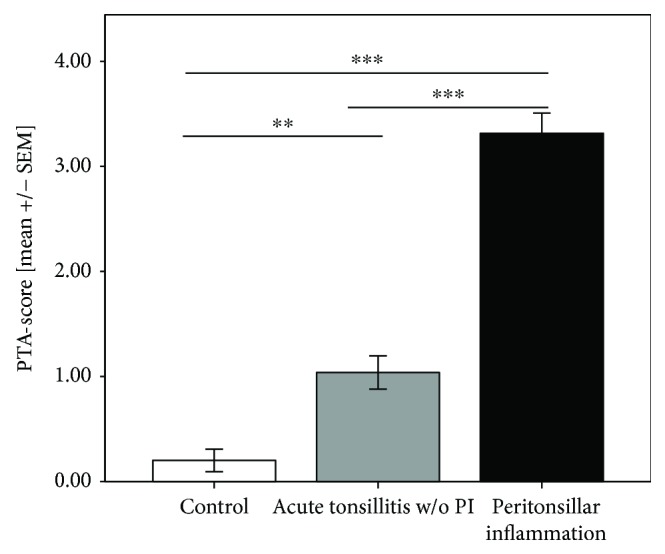
PTA score values. Patients with peritonsillar inflammation (PI) had significant increased PTA score values compared to controls and patients suffering from acute tonsillitis without (w/o) peritonsillar inflammation (^∗∗∗^*P* < 0.001, ^∗∗^*P* < 0.01).

**Figure 2:**
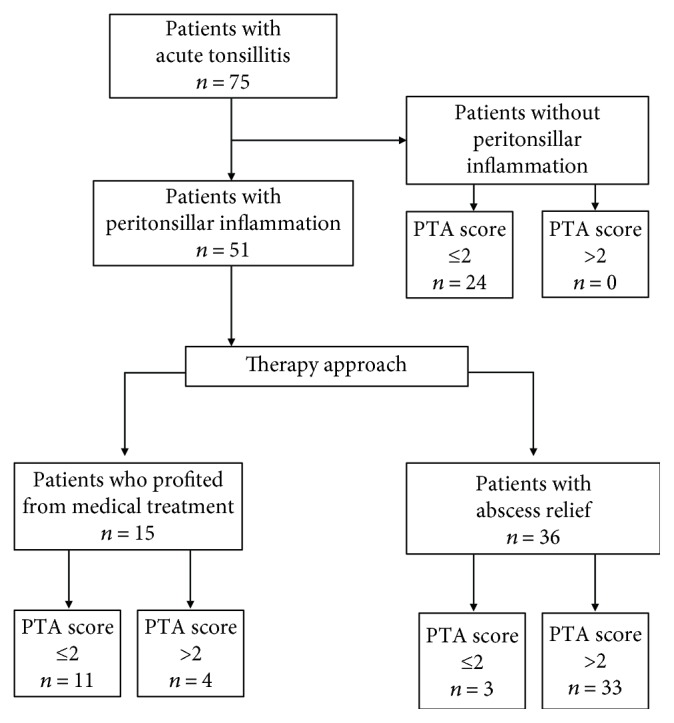
Patient flow diagram. Patient flow diagram showing the determined PTA scores of the patients with peritonsillar inflammation in dependence on the therapy approach.

PTA score values for each of the 91 subjects were determined. Both patients with (*S*_PTA_ = 3.35 ± 0.19) and without peritonsillar inflammation (*S*_PTA_ = 1.0 ± 0.15) showed significantly increased PTA score values in contrast to the healthy controls (*S*_PTA_ = 0.2 ± 0.11, *P* < 0.001, *P* = 0.001, resp.). Furthermore, the difference of *S*_PTA_ values between patients with and without PI was significant (*P* < 0.001) (Figure 1). 100% of the cases of the controls and patients without PI the PTA score values were *S*_PTA_ ≤ 2 (*n* = 40). Within the group of patients with peritonsillar inflammation, 72.5% of the cases showed *S*_PTA_ values > 2 (*n* = 37). *S*_PTA_ values ≤ 2 could be identified in the remaining 27.5% of the cases (*n* = 14) (Figure 2). Hence, the patients were further classified in two groups in dependence on PTA score values > 2 and ≤2.

The cohort with *S*_PTA_ values ≤ 2 showed a median age of 28 years (7–66 years) and consisted of 16 male and 23 female patients (*n* = 39). All patients received antibiotics, and 92.3% of the cases (*n* = 36) profited from medical treatment. In six cases (15.4%), an additional incision of the arched palate because of symptoms suspicious of PTA was proceeded but no pus was revealed. In 10.3% of the cases, tonsillectomy was performed (*n* = 4). Three of the patients who underwent tonsillectomy had an initial *S*_PTA_ value of 2 and showed a peritonsillar inflammation. Due to low response to medical treatment, abscess tonsillectomy was necessary in those cases. Furthermore, one of these three patients (16-year-old female) had a simultaneous infection with mononucleosis, and the further course was complicated by postoperative hemorrhage, two times. The fourth patient was a 23-year-old female with an initial PI and a *S*_PTA_ value of 0, but because of a mass suspicious of PTA in a computed tomography, tonsillectomy was performed. Intraoperatively, no abscess formation was detectable.

The age of the patients with *S*_PTA_ values > 2 (*n* = 37) was not different to the cohort with *S*_PTA_ values ≤ 2 (32 years (10–83 years) versus 28 (7–66 years), *P* = 0.36), and with 21 males and 16 females, the gender distribution was inverse. In 35 cases (94.6%), incision, needle aspiration, or tonsillectomy was performed. In 89.2% of the patients with *S*_PTA_ values > 2, pus was detected during the procedure. Four cases (10.8%) showed a good response to medical treatment. These cases had a mean *S*_PTA_ value of 4.25, and despite severe symptoms, incision revealed no pus in two of those cases.

### 3.2. PTA Score Values in Association with Different Procedures

**Figure 3:**
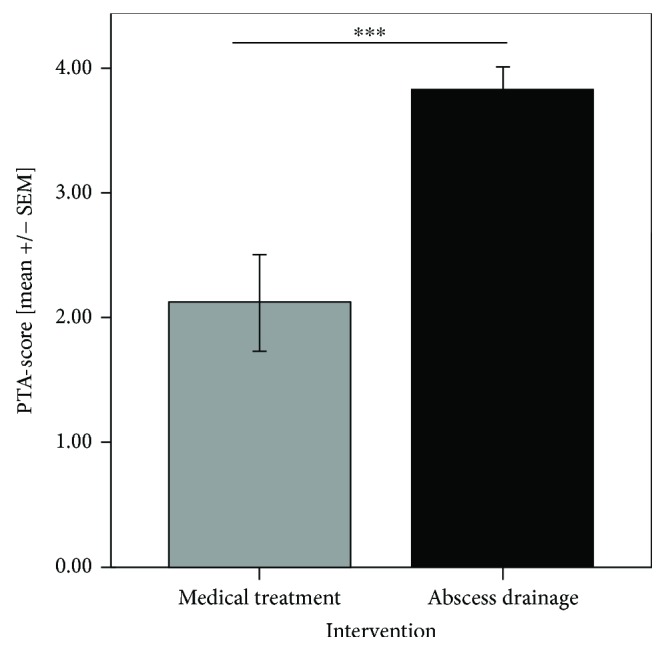
PTA score values of patients with peritonsillar inflammation. Patients who profited from medical treatment showed significantly reduced PTA score values compared to the patients with abscess relief (^∗∗∗^*P* < 0.001).

In total, 20 patients with peritonsillar inflammation refused incision, needle aspiration or suffered from severe symptoms which restricted the possibility of abscess relief in local anesthesia. Consequently, they underwent primary, immediate abscess tonsillectomy. However, 25 of the PI patients received a needle aspiration or incision in the area of inflammation because of an assumed abscess formation. In 44% of those cases, pus could be detected (*n* = 11), whereas no pus could be aspirated or relieved in the remaining 56% of the cases (*n* = 14). Retrospective analysis of these 14 cases revealed that eight patients benefited from further medical treatment, one patient underwent tonsillectomy, and in five cases, additional abscess tonsillectomy was performed due to severe symptoms of PTA. Six of the eight patients who benefited from medical treatment had a PTA score value ≤ 2, whereas four of the five cases with additional abscess tonsillectomy had an initial PTA score value >2. Interestingly, in the patient with additional abscess tonsillectomy and a PTA score value ≤ 2, pathologic examination of the tonsil revealed a coinfection with Epstein-Barr virus which was unique in the PTA group. Within the group of the eleven patients with pus aspiration or relief, all patients showed a PTA score value > 2. 63.6% (*n* = 7) of these patients profited from this procedure and further medical treatment. Just in four cases (37.4%), additional abscess tonsillectomy was necessary. In total, patients with peritonsillar inflammation who profited from medical treatment had significantly reduced PTA score values compared to the patients with abscess relief (2.4 ± 0.4 versus 3.8 ± 0.2, *P* < 0.001) (Figure 3).

### 3.3. Days of Hospitalization

Patients suffering from acute tonsillitis without concomitant peritonsillar inflammation benefited from medical treatment (100%, *n* = 24). If intravenous application of antibiotics was necessary, the patients were hospitalized for 4.0 days in median ranging from 2 to 8 days. Within the group with peritonsillar inflammation, the duration of hospitalization varied greatly in dependence on the procedures or treatment strategies which were performed. Patients who were treated medically had a median time of hospitalization of 5 days (range from 0 to 11 days), whereas patients who underwent abscess tonsillectomy stayed in hospital for 7.0 days in median ranging from 5 to 26 days. If the abscess could be relieved successfully by needle aspiration or incision, the median time of hospitalization was reduced to 4.0 days (range from 2 to 5 days). Compared to the group with PTA score values ≤ 2 (4.1 ± 0.4), the time of hospitalization is significantly increased in patients with PTA score values > 2 (7.3 ± 0.7, *P* < 0.001).

## 4. Discussion

With an estimated incidence of 30 people per 100,000 per year in the US, more than 11,000 hospital bed days and a mean duration of hospitalization of 6.2 ± 6.0 days (mean ± standard deviation), patients with peritonsillar abscess consume significant National Health Service resources [4, 16, 17]. Taken together, the patients with peritonsillar abscesses and peritonsillitis to the group of patients with peritonsillar inflammation, the incidence and associated costs of resources might be obviously higher. Treatment of patients with peritonsillar abscess commonly involves abscess drainage combined with antibiotic treatment or abscess tonsillectomy [3, 6, 18]. Generally, abscess drainage is performed via two common ways: needle aspiration or incision and drainage. Both procedures are invasive and were described to be painful but effective with a low to moderate rate of recurrences (incision and drainage: 4.7%, needle aspiration: 24.5%). There is no consensus concerning the best drainage method because each had special risks and benefits [6]. Furthermore, initial medical treatment has been described to be considerable in patients with less severe symptoms [5]. Despite the high incidence and importance of PI in otolaryngology services and primary care centers, there remains uncertainty concerning the differentiation between peritonsillitis and peritonsillar abscess and the individual therapeutic approaches [19–21]. To our best knowledge, this is the first report of an objective criterion to identify patients with peritonsillar inflammation who profit from medical treatment.

51 of 92 patients with a median age of 32 years ranging from 7 to 83 years showed a peritonsillar inflammation. 37 of these patients were characterized by *S*_PTA_ values > 2, and pus could be revealed in 33 of these cases. 14 patients with peritonsillar inflammation had *S*_PTA_ values equal or lower than 2, and in total, there were 36 patients with *S*_PTA_ values ≤ 2 who profit from medical treatment. A sensitivity of 91.7% and a specificity of 90.0% could be determined for the PTA score to identify patients who profit from medical treatment with a negative predictive value of 0.92 and a positive predictive value of 0.89. However, the sensitivity and specificity of the PTA score may be restricted in cases with some specific pathogens, like *Epstein-Barr virus* and *Mycobacterium tuberculosis*, or in patients with compromised immunity and infection with opportunistic pathogens like *Candida*. Further studies with larger cohorts are necessary to analyze the potential of the PTA score in those special cases.

**Figure 4:**
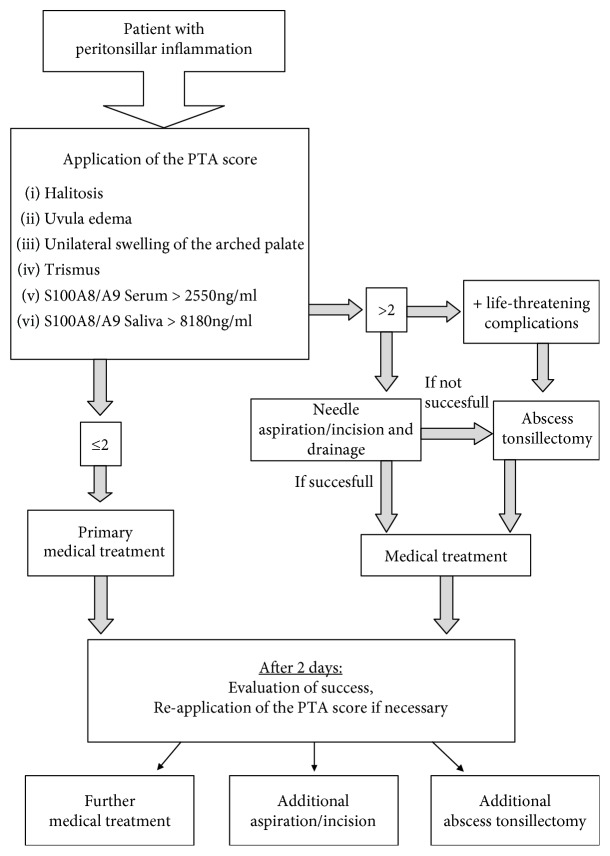
Therapy flow diagram. Diagram of the suggested algorithm with application of the PTA score determining the therapeutic approach.

We could show the PTA score to be an appropriate tool to determine the indication for needle aspiration or abscess incision. Due to a high probability of peritonsillar abscess existence, needle aspiration or incision and drainage should be performed in patients with PTA scores > 2 to avoid abscess tonsillectomies and their associated complications like postoperative hemorrhage. Patients with peritonsillar abscess who profit from initial abscess relief in local anesthesia had a significantly reduced time of hospitalization compared to the patients with abscess tonsillectomy (3.7 ± 0.8 versus 8.3 ± 0.7, *P* = 0.004). The increased length of stay in hospital results from an extended time of observation because of the risk of postoperative hemorrhage [7]. Furthermore, female gender, advanced age, and comorbidities like diabetes or hypertension have been described to be negative predictors concerning the time of hospitalization [4]. Accordingly, the duration of hospitalization and consequently the costs of the treatment of patients with peritonsillar inflammation can be reduced if decisions concerning the treatment strategy are based on the PTA score. Several reports support the outpatient management of PTA which is common routine practice in the US [22, 23]. However, this recommendation is of a low evidence and an objective, reliable instrument to differentiate whether in- or outpatient management is required remains desirable [17]. The PTA score is a helpful tool to determine the optimal treatment approach, and further studies have to examine its value to identify the patients who profit from inpatient treatment strategy. Additionally, incipient application of the new PTA score reduces the amount of gratuitous, painful needle aspiration or incision, and the probability of correct treatment with these procedures increases from 44% to 64.7%. In cases of patients with peritonsillar inflammation, we suggest a proceeding as shown in Figure 4. For acquisition of S100A8/A9 levels in serum and saliva, a rapid and quantitative assay has to be defined in future which is easily executable during outpatient consultation. Although we were not able to certainly exclude an abscess formation in patients with PC, we could demonstrate the PTA score's value as an appropriate tool to identify patients who profit from medical treatment. Nevertheless, further prospective, randomized clinical trials with bigger cohorts are necessary to elucidate high-quality evidence, sensitivity, specificity, and accuracy of the PTA score as an objective criterion to identify patients benefiting from medical treatment.

## 5. Conclusion

The PTA score is an appropriate tool to identify patients with peritonsillar inflammation profiting from medical treatment. Application of the PTA score helps to determine the treatment strategy, avoids unnecessary procedures, and reduces the time of hospitalization.

## Figures and Tables

**Table 1:** Patient characteristics due to diagnosis. Values of significance by Kruskal-Wallis test. d, days; PTA, peritonsillar abscess; y, years.

Variable	Frequency, number (%, range)			*P* value
	Acute tonsillitis	Peritonsillitis	Peritonsillar abscess	
Age, median (range), y	25 (13–65)	32 (7–66)	32 (10–83)	0.150
Sex				0.860
Male	11 (44)	7 (47)	19 (53)	
Female	13 (56)	8 (53)	17 (47)	
Therapy approach				<0.001
Only medical treatment	24 (100)	6 (40)	0 (0)	
Incision/aspiration	0	9 (60)	16 (44)	
Tonsillectomy	0	1 (7)	29 (81)	
Hospitalization, median (range), d	4 (2–8)	5 (0–11)	7 (2–26)	<0.001
PTA score value				<0.001
≤2	24 (100)	11 (73)	3 (8)	
>2	0 (0)	4 (27)	33 (92)	

## Data Availability

The datasets used to support this study are currently under embargo while the research findings are under further investigation. Access to these data will be considered by the corresponding author upon reasonable request.
